# Disclosure of true medical information: the case of Bangladesh

**DOI:** 10.1186/s12910-024-01115-y

**Published:** 2024-10-17

**Authors:** Sanwar Siraj, Kristien Hens, Yousuf Ali

**Affiliations:** 1 Centre for Medical Ethics and Law, Medical Ethics and Humanities Unit, The University of Hong Kong, Pok Fu Lam, Hong Kong SAR; 2 Department of Philosophy, University of Antwerp, Antwerp, Belgium; 3 Department of Political Science, Dhaka International University, Dhaka, Bangladesh

**Keywords:** Medical ethics, Health law, Truth-telling, Phenomenological study, Collective society, Bangladesh

## Abstract

**Background:**

Truth-telling in health care is about providing patients with accurate information about their diagnoses and prognoses to enable them to make decisions that can benefit their overall health. Physicians worldwide, especially in the United Kingdom (U.K.) and the United States (U.S.), openly share such medical information. Bangladesh, however, is a Muslim-majority society with different social norms than Western societies. Therefore, we examined whether Muslim culture supports truth disclosure for patients, particularly how and to what extent medical information about life-threatening diseases is provided to patients in Bangladesh.

**Methods:**

This was a phenomenological qualitative study. We conducted thirty in-depth interviews with clinicians, nurses, patients and their relatives at Shaheed Suhrawardy Medical College Hospital in Dhaka, Bangladesh. We also used observations to explore interactions between patients, families and healthcare professionals regarding their involvement in medical decisions and truth disclosure issues. NVivo software was used to identify common themes, and a thematic analysis method was utilised to analyse the datasets.

**Results:**

This study identified three recurring themes relevant to the ethics and practice of truth disclosure: best interest rather than autonomy, the ambivalent value of deception and who understands what. The participants revealed that physicians often withhold fatal medical prognoses from terminally ill patients to ensure the best healthcare outcomes. The results indicate that deception towards patients is commonly accepted as a means of reducing burden and providing comfort. The participants opined that true medical information should be withheld from some patients, assuming that such disclosures may create a severe burden on them. Whether or to what extent medical information is disclosed primarily depends on a family’s wishes and preferences.

**Conclusions:**

While truth disclosure to patients is considered an ethical norm in many cultures, such as in the U.K. and the U.S., the practice of concealing or partially revealing severe medical prognoses to patients is an actual medical practice in Bangladeshi society. This study emphasises the importance of recognising a patient’s active involvement and respecting the cultural values that shape family involvement in medical decision-making. These findings may have significant policy and practical implications for promoting patient autonomy within Bangladeshi family dynamics and religious-based cultural values.

**Supplementary Information:**

The online version contains supplementary material available at 10.1186/s12910-024-01115-y.

## Background

Bangladesh’s healthcare system is pluralistic, with four key sectors – public, private, non-governmental organisations (NGOs) and international donor agencies – that shape its structure and function [1]. Constitutionally, the government of Bangladesh is committed to providing primary healthcare services and associated logistics to its citizens via publicly funded hospitals [2] and with the majority of the government’s budget allocated to these hospitals. However, the government faces challenges in delivering basic healthcare services to the vast majority of its population, as only 2.36% of Bangladesh’s gross domestic product (GDP) is spent on healthcare [3]. This figure is even lower than that of other socioeconomically similar countries in South Asia. For example, Nepal spends approximately 5.42% of its GDP on healthcare [4], while the Maldives spends over 10% [5].

Bangladesh’s public healthcare system operates on a highly centralised, top-down approach, with the Ministry of Health and Family Welfare responsible for planning and proposing health policies [6]. Public healthcare services are delivered at the national, divisional, district, subdistrict, union and ward levels [7]. National and divisional hospitals in Bangladesh offer tertiary-level healthcare facilities. District hospitals, integral to the public healthcare system, provide specialist services in addition to primary care. The Upazila (subdistrict) Health Complex delivers first-referral (secondary) care, including comprehensive emergency obstetrical care. Union Health and Family Welfare Centres and community clinics at the ward level across the country focus on delivering primary healthcare services.

Public hospitals provide care free of charge or at subsidised rates, while private hospitals primarily operate for profit [8]. Although affluent patients tend to prefer private hospitals due to their reliably high standards of diagnosis, care and treatment, most Bangladeshis, particularly from low- and low-middle-income groups, rely primarily on government-funded hospitals [9, 10]. The number of hospitals is insufficient to meet demand, resulting in public hospitals and clinics being consistently busy and overwhelmingly crowded [11].

Bangladesh is a majority Muslim society in the South Asian region, with a population of 171 million [12], approximately 91% of whom adhere to Islam [13]. While the country constitutionally recognises Islam as its official religion, people of other faiths can practice their beliefs in peace and harmony [2]. Islamic ethical principles guide the everyday lives of Muslims, including healthcare decisions [14].

The family is the fundamental unit of Bangladeshi society [15]. Each individual is born, grows up and lives within the family. For most Bangladeshis, his or her family is the core of daily life. While socioeconomic factors drive adult children to relocate, particularly from rural to urban areas, many still maintain the tradition of living together in extended or ‘joint’ families. These households often encompass three or more generations living under one roof or nearby [16]. It is customary for sons to remain with their parents after marriage, fostering robust intergenerational bonds vertically and horizontally. Although married daughters typically move in with their husbands’ families, their connections to their own families of origin remain strong.

Family members cooperate in familial matters rather than confront each other. In the lives of Muslims, no activity is considered purely secular. Maintaining good relationships with family members is a societal norm and religious duty (The Holy Quran, 13:21). These duties include looking after family members and providing support in times of need. This religious and ethical approach can help Muslim Bangladeshi families make the necessary decisions regarding healthcare treatment for their loved one.

In many collective societies, health issues are viewed as ‘family affairs’ [17], with religion playing a significant role in shaping the meanings of illness, suffering, death and preferences for healthcare decisions as well as end-of-life care treatment [18–21]. What is considered morally right or wrong can vary depending on the specific sociocultural context in which healthcare decisions are made [22]. Muslim families may often choose to keep a life-threatening diagnosis or prognosis confidential, especially for elderly patients with terminal illnesses, to prevent further burdens [23].

Physicians in many traditional cultures may also be asked to disclose severe medical prognoses to a family and to withhold them from a patient [24–26] or to carefully disclose information to the patient when they can handle it [27]. While the Western ethical norm, mostly relevant in the United Kingdom (U.K.) and the United States (U.S.), is that patients should be informed about their healthcare, this perspective may not be universally shared in practice [21]. This study investigated the ethical norms and actual practices of truth disclosure within the context of Bangladeshi society and culture.

Despite the absence of specific laws and regulations on truth-telling practices, the Code of Professional Conduct, Etiquette and Ethics of the Bangladesh Medical and Dental Council (BMDC) mandates that healthcare professionals inform patients about all aspects of their clinical management, including examinations and investigations [28]. The Code encourages physicians to deliver medical opinions with the utmost clarity to ensure that patients and their relatives understand them fully.

The practice of truth-telling in medicine is often influenced by cultural and religious factors [29]. This paper examined how societal ethos, cultural norms and religious ethics impact practices related to the disclosure of medical information, particularly for patients diagnosed with severe diseases in Bangladesh. This study’s objective was to explore the disclosure of information related to a patient’s medical diagnoses and treatments and the parties to whom they are disclosed. However, the extent of the involvement of families, patients, clinicians and caregivers in the truth-telling process remains unknown. It addressed this gap by exploring several ethical questions:

- How are patients informed about and asked for consent for medical interventions? What happens when a patient is diagnosed with a lethal prognosis?
- Who bears the responsibility of disclosing medical information to patients, families or physicians?
- To what extent is information provided to whom and on what basis?

## Methods

### Study setting

This is a phenomenological qualitative study [30]. This study design is well suited for this study because it primarily focuses on exploring the lived experiences and practical perspectives of individuals. In this current case, it involved physicians, nurses, patients and their relatives involved in the practice of medical decision-making, particularly disclosing true medical information to patients diagnosed with severe diseases in Bangladesh. Using phenomenological qualitative methods, such as interviews and observations, this study delved deeply into the experiences, perceptions and meanings attached to true disclosure, providing rich and detailed insights that can inform decision-making issues in healthcare settings. A phenomenological framework helped us uncover the patterns and complexities of true medical disclosure practices within Bangladeshi religious culture and family-based society.

This study was conducted at Shaheed Suhrawardy Medical College (ShSMC) Hospital, which is located in the capital city of Dhaka, Bangladesh. ShSMC is a publicly funded hospital that provides tertiary-level care. Equipped with 850 indoor beds, it has thirty-five departments that provide modern medical care.

ShSMC Hospital was selected because it provides quality treatment and trustworthy care to many patients daily. For example, on average, 2,000 patients receive outpatient services from different departments. About 250 patients receive daily admissions to different inpatient departments, and 300 patients use emergency services from this hospital’s emergency department. This hospital is considered representative, as most patients regularly seek healthcare services at public hospitals for free or subsidised costs. Moreover, it was selected as a study site because of its renowned and experienced clinicians and healthcare staff.

### Study participants

A purposive sampling method [31] was used to recruit research participants who could provide rich insights into the disclosure of true medical prognoses and diagnoses for patients diagnosed with terminal illnesses. The selection criteria included collecting evidence from patients, family members, and healthcare professionals at ShSMC Hospital who were over eighteen years of age and who voluntarily participated. This study collected empirical evidence from thirty participants (eighteen males and twelve females), including five expert physicians, five senior staff nurses, and ten patients, and ten family members at different inpatient and outpatient departments/units of ShSMC Hospital (see Table 1).

All the participants were recruited exclusively for this study, and there was no prior interaction between the participants and the research team except for announcements regarding the interviews. While both male and female participants were included in each group, only male patients were interviewed due to religious sensitivity regarding male interviewers entering inpatient departments/units to interview female patients. All of the nurses interviewed for this study were female, reflecting the predominance of female nurses in the hospital. One interviewed physician is a female who worked as the head of the Obstetrics and Gynaecology Department, while the other four doctors are men. Of the ten family members who participated in this study, six were female. The question-and-answer session was conducted on a one-to-one basis.

**Table 1:** Participants’ demographic and medical information

Participant	Role	Age	Gender	Specialisation (for the nurses/doctors)	Affliction	Education Level	Income Status
D1	Physician	59	Female	Obstetrics and Gynaecology		PhD	High
D2	Physician	25	Male	Physical Medicine		MBBS	Middle
D3	Physician	38	Male	General Medicine		Fellow of College of Physicians and Surgeons (FCPS: in progress)	Middle
D4	Physician	55	Male	Obstetrics and Gynaecology		FCPS	High
D5	Physician	35	Male	Urology and Nephrology		FCPS (in progress)	Upper-middle
N1	Nurse	45	Female	Urology		Undergraduate	Upper-middle
N2	Nurse	38	Female	Endoscopy		Diploma	Middle
N3	Nurse	35	Female	Neuro-Medicine		Diploma	Middle
N4	Nurse	55	Female	ENT		Undergraduate	Middle
N5	Nurse	37	Female	General Medicine		Diploma	Middle
P1	Patient	63	Male		Third heart attack	Higher Secondary Certificate	Middle
P2	Patient	35	Male		Tumour infection	Grade 7	Low
P3	Patient	34	Male		Fistula	Higher Secondary Certificate	Upper-middle
P4	Patient	32	Male		Gallstone	Undergraduate	Upper-middle
P5	Patient	42	Male		Fistula	Master’s	Middle
P6	Patient	28	Male		Fistula	Higher Secondary Certificate	Middle
P7	Patient	58	Male		Tumour and fistula	Master’s	Upper-middle
P8	Patient	30	Male		Corneal surgery	Illiterate	Low
P9	Patient	39	Male		Gallstone	Undergraduate	Middle
P10	Patient	62	Male		Spine surgery	Grade 9	Low
R1	Family member	35	Female		Appendicitis	Grade 5	Low
R2	Family member	28	Male		Heart attack	Master’s	Upper-middle
R3	Family member	42	Female		Kidney	Undergraduate	Low
R4	Family member	20	Female		Eye Operation	Higher Secondary Certificate	Low
R5	Family member	30	Male		Legs (gangrene)	Hafaz-e Quran (Memoriser of the Quran)	Low
R6	Family member	35	Female		Cancer	Higher Secondary Certificate	Middle
R7	Family member	38	Female		Cancer	Masters	Middle
R8	Family member	48	Male		Hysterectomy	Masters	High
R9	Family member	29	Male		Fistula	Secondary School Certificate	Low
R10	Family member	30	Female		Lung infection	Secondary School Certificate	Middle

### Study design

Semi-structured, in-depth interviews [32] were conducted to determine how and whether patients’ medical prognoses had been disclosed. We obtained a list of physicians and nurses and their short biographies working in the hospital. We then approached clinicians and nurses who are particularly experienced and who provided regular care to patients at the hospital.

Our goal in using the purposive sampling technique was primarily to gather narratives and lived experiences from patients and their relatives as well as experienced physicians and nurses who regularly provide care to patients across various inpatient and outpatient departments within the hospital. We collected a list of patients admitted to the hospital’s indoor units. Patients typically visit hospitals accompanied by their relatives.

The family members involved in this study were close relatives of the patients and, in some cases, extended relatives. These family members often stay with their loved ones at the hospital, providing care and support during hospitalisation. We approached patients and family members during their free time about their willingness to participate in this study.

A set of open-ended Bengali-version questionnaires (with 98% of Bangladeshis using Bengali as their first language) was used, which enabled us to explore the diverse insights of the selected participants [33]. An English version of these interview questions is attached to this manuscript (see Additional File 1). The participants received no compensation for participating.

Interviews with patients and their relatives and with nurses were conducted entirely in Bengali, while conversations with physicians were conducted in English. Nurses and clinicians were individually interviewed in their offices, while patients and their relatives were interviewed in quiet places on the hospital premises. The interviews were recorded using a tape recorder, and observational notes were kept as supplements. Each interview lasted 30–40 min.

We also closely observed how patients, families and clinicians interacted while discussing what kind of medical information was disclosed and to whom. We employed ‘direct’ observation for this study because it aided in reducing potential bias. We also developed a structured checklist that helped us minimize subjective biases in our observations. We took field notes from the discussions and stories from each observation. A large amount of time—from morning to evening, three times per week—was spent observing particularly how families consulted with physicians about whether and to what extent medical information was provided to patients if they were diagnosed with terminal diseases. This study observed twelve cases in total, with each observation lasting 10–20 min. The empirical fieldwork for this study began in mid-October 2022 and continued over six weeks.

### Data analysis

The research team consisted of three members. One member, Yousuf Ali (YA), conducted interviews and observations. YA transcribed audio recordings and observational notes into Bengali and then translated them into verbatim English for analysis. YA graduated from a publicly funded university in Bangladesh and has extensive experience conducting empirical fieldwork in healthcare settings. Sanwar Siraj (SS) and Kristien Hens (KH) utilised NVivo software to analyse the datasets. This software helped uncover underlying common themes with appropriate interview and observational notes. An inductive approach was used to guide this study [34].

Subsequently, a thematic analysis method [35] was used to present the results of this study along with participants’ quotes. SS and KH performed initial coding that stayed close to the text. Then, SS and KH combined these codes into broader categories. For anonymisation purposes, codes were given to the participants: physicians were coded as D (1–5), staff nurses as N (1–5), patients as P (1–10) and relatives as R (1–10).

The themes were related to the research questions. The results were then summarised and presented using one or more sentences or paragraphs. SS and YA then compared observational field notes with data from the interviews to identify and validate themes. Once our analysis was complete, SS and KH exported the findings, including coded data for reporting purposes. The themes presented in the Results section were agreed on by all of the authors (SS, KH and YA).

### Informed consent and ethics approval

This study was conducted according to the ethical principles of the World Medical Association Declaration of Helsinki [36]. It was vetted by the Institutional Ethics Review Committee of ShSMC Hospital (no. ShSMC/2022/042), Dhaka, Bangladesh. Informed consent was obtained from all study participants. The study protocol, including the interview guidelines, was written in Bengali. Before obtaining informed consent, we thoroughly explained orally and in writing to each participant the purpose of this study, inclusion and exclusion criteria, potential risks and benefits, the withdrawal process and research outputs. We also emphasised the importance of privacy and confidentiality, informing the participants that anonymous codes would be used during the data analysis to protect their identities. All other personal information was also anonymised for analysis and reporting.

## Results

This paper is part of a larger study that focuses on issues related to truth disclosure within medical settings in Bangladesh. This section reports the findings from thirty in-depth interviews and observations conducted during the field visits. It identifies key themes relevant to the research question: How do patients, family members, nurses and physicians in Bangladesh perceive the disclosure of true medical information? This study identified three key themes to address this question: ‘best interests rather than autonomy’, ‘the ambivalent value of deception’ and ‘who understands what’.

### Theme 1: best interest rather than autonomy

Narrative highlights prioritising patient well-being over autonomy in the context of truth disclosure in healthcare. It is predominantly believed that physicians should prioritise patients’ best health interests over their right to make decisions for themselves. One interviewee suggested that healthcare professionals aim to balance the ethical principles of beneficence, non-maleficence and patient autonomy. A health staff member acknowledged that disclosing the complete truth may not always align with a patient’s best interests. The interviewee believed that withholding information may be more beneficial for protecting a patient’s well-being.

In situations where disclosing the truth could be particularly distressing, such as terminal diagnoses or severe diseases, such as cancer, physicians tend to first discuss this information with a patient’s family:

If a patient is diagnosed with malignancy (a type of cancer), we do not tell the patient directly. We call for a discussion with the patient’s family. (D1)

I was diagnosed with severe heart problems, but the doctors did not inform me about my health issues. Instead, they informed my wife and nephew. (P1)

We prioritise the patient’s best interest by choosing not to inform them about the nature of their disease. Instead, we believe it is more beneficial to communicate this information to their family members. (N5)

Our observations revealed that physicians seek consent and decisions from family members regarding the disclosure of information to patients. In cases where patients express a desire to know their medical prognoses for severe conditions, physicians often choose to use ‘vague’ terms. The physicians explained that they opt for vague terms to prioritise patients’ best interests rather than strictly adhering to the principle of patient autonomy in knowing true information.

When the physicians were asked why they used vague terms when disclosing severe medical prognoses to patients, they mentioned that they did this to prevent potentially severe health consequences that may arise from full truth disclosure. One family member said that sharing the complete truth could lead patients to feel hopeless. One physician admitted to engaging in discussions with families to determine the extent to which certain information should be shared with patients.

The interviewees emphasised the importance of carefully considering how and to what extent information should be disclosed to patients in such situations:

If the families allow us, we then disclose it to patients directly, or in some cases, families disclose it to their patients gradually. If we are allowed to disclose, we disclose it slowly, and we use a vague term such as ‘tumour’ or other words. (D1)

If patients want to know their prognosis, physicians disclose it using some other ‘vague’ terms. (R1)

As family members, we chose not to inform our patient of the true prognosis out of concern that it might cause fear and increased stress, shifting the focus onto us and our family rather than the patient’s physical condition. We believe that discussing life-threatening diseases directly with patients may potentially exacerbate their condition. (R7)

When asked why clinicians do not directly disclose a true medical prognosis to patients, the participants expressed the view that patients with severe diseases should not be fully informed about the medical prognosis. The clinicians emphasised that the decision to withhold certain information from vulnerable patients, especially those with limited education and awareness, is solely about prioritising saving their lives and ensuring their well-being rather than protecting their autonomy.

They acknowledged that many individuals reside in rural areas, with a sizeable portion having limited education. Approximately 60% of the population in Bangladesh lives in rural regions [37] with a majority having minimal to no formal education. For instance, according to the World Bank’s 2021 data, only around 38% of Bangladesh’s population aged 25 and over has completed at least upper secondary education [38]. Many people in these areas have a limited understanding of medicine as well as the nature of diseases and their severity. Our observations revealed that if a patient is diagnosed with a severe illness (e.g., cancer) and is informed of it directly, they may struggle to cope with the sad news, leading to increased emotional pain and psychological distress.

The clinicians’ views align with our observations:

We are cautious about directly disclosing all information to patients in cases of serious medical issues. Full disclosure of details regarding a major illness can potentially have grave consequences for the patient. (D3)

Many patients and their families residing in rural areas lack awareness about health and diseases. For individuals with limited education and knowledge, families often fear that disclosing certain information may lead patients to experience extreme stress, potentially exacerbating their condition. In situations where the patient is unconscious, families may believe that it is inappropriate to share such information. (D5)

Our question pertains to what motivates clinicians to disclose accurate medical diagnoses and prognoses to families instead of directly to patients. By observing interactions among families, patients and healthcare professionals, it was noted that financial considerations often drive clinicians to reveal a true medical prognosis to families rather than to patients themselves.

Additionally, our observations indicate that whether a patient diagnosed with a severe disease should be informed often hinges on his or her family’s preferences. Families are typically responsible for making decisions regarding the care and treatment of their loved ones, including arranging for the payment of medical services. Patients place trust and confidence in their family members’ decisions.

The views expressed by family members further support our observations:

If a patient is not able to give consent, consent is obtained from the family. The family is called upon to give consent on financial matters. (R2)

Since the family takes responsibility for the patient, the family does not need to inform the patient about the disease. The patient feels comfortable with the family’s decisions. (R3)

Our inquiry focused on why families request that clinicians not disclose severe medical prognoses directly to patients. Our observations revealed that physicians often align with a family’s preferences when they believe it is in the “best interest” or beneficence of the patient. Physicians recognise a family’s intimate knowledge of a patient and trust their ability to assess the patient’s psychological state and capacity to handle certain information. Families aim to prevent overwhelming their loved ones and prioritise facilitating a peaceful end, especially for elderly patients, emphasising the significance of a peaceful dying process for the patient.

These observations supported the narratives provided by the interviewees:

The patient is given as much information that is comfortable; they can tolerate as much as possible. (D3)

Families know their patients well. Families are aware of their patients’ information levels of tolerance. (N4)

Doctors give hope to patients. Families also do not want to disclose it to their patients if the elderly are for peaceful dying. (R1)

Our inquiry into why patients are not informed about their true prognoses revealed that withholding information is often justified by family members who believe their loved ones may lack the capacity to make well-informed medical decisions on their own.

The respondents emphasised the importance of patients being embedded within familial structures, where family members are viewed as having a deep understanding of everyone’s needs. One family member questioned the rationale behind directly disclosing medical prognoses to patients, suggesting that the purpose may not solely be for patients to make medical decisions alone.

The family unit was regarded as a protective space where the best interests of each patient were safeguarded:

The doctor and our family jointly make the decision. It is understandable that those outside the family may not prioritise ensuring the patient’s best interests. R4.

Family decision-making may affect patient autonomy. But the family has some responsibilities. (P6)

The culture of our country is to tell the family everything. Not telling the patient about major problems. (N1)

### Theme 2: the ambivalent value of deception

This theme explores narratives and observations of how the deception of true medical information and prognosis can have both positive and negative implications. We delved into instances in which lying may be justified in certain circumstances while recognising the associated risks and ethical considerations linked to deception in the medical context.

Our data revealed that patients often refrained from telling the truth about their true prognoses when diagnosed with a severe disease. We observed instances where families and clinicians informed their patients that nothing significant had happened to their health and that medicine may provide a cure. Interviewees opined that deception is sometimes okay to provide safe care and prevent additional anxiety. The interviewees expressed that deception is not a negative approach:

The doctor told me differently. They did not tell me the truth, as I think it may have caused me panic. (P2)

We, as doctors, use different terms when communicating with patients diagnosed with severe diseases such as cancer. We do this in such a way that there is a possibility that the patient may experience severe psychological burdens. (D3)

Our observations shed light on a common occurrence in which families ask clinicians to employ deception in their communication with patients. In these situations, families explicitly instruct physicians not to disclose the true medical prognosis directly to patients, citing a concern for mitigating or avoiding potential harm (non-maleficence). This directive frequently emanates from a genuine desire to shield patients from distressing information that may have adverse effects on their emotional well-being. The motivation behind this practice is rooted in the belief that by withholding specific details, clinicians and families can protect their loved ones from unnecessary anxiety and the potential threat of dying. Families perceived this approach as contributing to the creation of a more supportive environment during medical treatment. Deception is accepted to comfort patients, minimising burdens on patients’ health.

Interviewees shared their experiences accordingly:

There is deception. It happens when there is a major disease like cancer. (P3)

We do not immediately disclose anything harmful to any patients or their families because they may not tolerate the crucial negative health information if the patient is diagnosed with severe diseases, such as cancer. Patients may not bear such bad health news, and it may negatively impact their health, both psychologically and physically. (D1)

If the patient is told immediately, his or her physical condition may worsen. Many patients also commit suicide. (D4)

Everyone wants to survive. If one comes to know about a disease, such as dying, then one may become afraid and create worse conditions. That is why the patient’s family is informed, and the patient is not always told the truth. The patient’s family forbids the doctor from discussing the major illness with the patient. (R4)

Our observations indicated that clinicians and health staff deceive patients and refrain from disclosing true medical information to reduce their psychological distress and provide compassionate care. It was noted that patients often experience emotional relief and comfort when engaging in conversations with their doctors and receiving messages of hope from healthcare providers. Such expressions of hope can contribute to the psychological well-being of patients, fostering a sense of optimism and potentially aiding in their recovery process.

A nurse (N3) shared that her role involves providing care for the psychological well-being and emotional resilience of patients diagnosed with severe diseases. She stated that the true medical prognosis is withheld from patients to lessen distress and provide emotional support. Two interviewees emphasised the importance of this approach.

I underwent heart surgery and was given rings. I was not fully aware of my health condition, but my family members were informed. Neither the physicians nor my family disclosed all the details to me to prevent me from becoming anxious. I have recently been informed that I need another heart surgery. Initially, doctors and my family assured me that nothing was wrong with my health…Whether it is considered deception or complete concealment of information, it is acceptable. Otherwise, I might suddenly become nervous and may not be able to cope psychologically. (P1)

The doctor initially informed me that my father had a stomach tumour but assured me that it was not a cause for concern. The doctor also expressed hope to me that patients with this type of condition typically show improvement following surgery. (R9)

Our observations also reveal perspectives regarding the communication of patients’ true prognoses. Interviewees stated that deception may have a negative impact. Patients often understand their true health conditions and engage with their families in making important healthcare decisions. Some believe that deception may hinder patients from making their own treatment decisions. Some opined that deception is an inappropriate medical practice. They argued that deception might be viewed as non-beneficial and could discourage patients from knowing their true reality, thereby preventing them from actively participating in their healthcare journeys.

Others stated that transparency in communicating a true medical prognosis is essential, as it may foster trust between clinicians and patients and facilitate patients in making informed decisions, thereby contributing to the patient receiving proper treatment.

Patients should be informed so that they stick to the treatment plan. (N2)

It is better to make patients’ own decisions. However, this is not the case in all cases. It is better to let the patient know to make decisions alone. If there is no fear or deception and the patient can decide alone, it will result in a good outcome for the patient’s health. (P6)

Patients are more aware of their health and diseases. If true information is disclosed to patients, it may help them make appropriate and proper treatment decisions. I had stones in my gallbladder, and I was informed directly. It was easy for me to make prompt decisions about having surgery. (P9)

### Theme 3: who understands what?

This theme highlights how healthcare professionals effectively convey accurate, comprehensive medical information to patients, ensuring their understanding and facilitating proper decision-making for a patient’s treatment. Our observations indicate that healthcare professionals frequently face ethical dilemmas when determining how much information to disclose to patients about their conditions, treatments and prognoses.

Physicians often grapple with the question of who should be informed. A decision about who should be informed depends on the clinicians’ understanding of who can make the best decisions for the patients. Our observations reveal that minor patients under the age of eighteen are not directly informed about severe medical prognoses for their illnesses. This cautious approach is derived from physicians’ understanding that minor patients may lack the cognitive capacity to make well-informed medical decisions regarding their health.

The physicians stated that conveying medical information to minor patients’ relatives may result in better outcomes. Families of minor patients may be better equipped to make decisions for patients. This precautionary measure is implemented to safeguard against potential adverse consequences that could arise from minors making treatment decisions without the necessary understanding or maturity.

If there is any health problem with a minor patient, we don’t tell them directly; instead, we provide the full information to the family of the minor patient. (N3)

When minor patients under eighteen years of age come to us, we tell their guardians all the medical information. Talking to a minor patient with this condition is not useful. Despite hearing from a minor patient what the disease is, what the problem is and what has been tested, we usually discuss it with the parents of minor patients. Minor patients who come to us for treatment must be accompanied by parents or guardians. (D4)

If a patient is a minor, doctors inform his or her family about the disease. Minors cannot make decisions themselves. So, whether to tell a minor patient about their condition amounts to the same thing. (P4)

Our observations underscore a notable trend in which patients who are educated exhibit increased awareness and conscientiousness regarding their health. Specifically, we observed that patients, particularly those with a higher level of education, are often more actively engaged in their healthcare journeys. These educated patients frequently ask questions of their families and clinicians to better understand their health conditions and medical prognoses. Interviews and observations revealed that patients with higher education levels tended to better understand their health conditions. Respondents suggested that if an educated patient is diagnosed with a terminal illness, they often understand the severity of their condition by reviewing their medical reports.

Physicians acknowledge that disclosing the truth may not place an undue burden on patients who are well educated and have knowledge of medicine. For instance, three patients (P5, P7 and P9) interviewed in this study are well educated. When questioned, these patients indicated that they were aware of their illnesses and understood the gravity of their situation by interpreting their medical reports. Insights from the interviewees further corroborate these observations, highlighting the link between educational attainment, economic stability and a patient’s ability to navigate their healthcare decisions with an understanding of the clinician’s explanations.

Education helps patients understand the nature of the disease. (R6)

If a patient is educated, then they can understand everything when they see the diagnostic results. (P2)

If a patient is educated, s/he is gradually informed about everything because educated patients can understand their illness and its severity. (D3)

Another aspect of this theme involves healthcare professionals’ understanding of and decision-making processes regarding truth-telling. Clinicians mentioned that they disclose the true medical prognosis to their patients in exceptional cases when it is necessary for them. Our field observation particularly indicates that physicians disclose medical information or prognoses to patients in exceptional cases to prevent fear in unmarried but pregnant adult girls.

Physicians understand that the life of a pregnant but unmarried girl may become riskier if they do not disclose the true prognosis. Once unmarried girls know they are pregnant, they request that clinicians not reveal the true medical prognosis to their families. Physicians respond to patients’ requests and provide medical interventions as needed. They avoid disclosing such distressing news to the families of pregnant women to protect vulnerable patients.

One physician who practiced obstetrics and gynaecology and a staff nurse shared their extensive experience as follows:

The patient’s family is totally responsible for the treatment decision. However, we have some exceptions; for example, there are some issues and matters that we keep secret from families. We keep it within ourselves and our patients. For example, if an unmarried adult girl becomes pregnant, it is illegal in our culture, and we do not disclose these secret issues to patients’ families. These patients requested that we hide this information from their families. Such an unmarried girl is afraid to disclose such shocking news to their families and ask us to hide this information. (D1)

If it is a sensitive issue for the patient, we take time to understand it. This information is not shared with others. We do this in the best interests of the patient. (N1)

## Discussion

A key finding of this study is that clinicians did not disclose severe medical prognoses or information to patients diagnosed with terminal illnesses. The participants indicated that families typically make decisions for patients and believe that families should be informed about sad news regarding patients with severe prognoses. One of the underlying reasons for this indigenous approach is the vital role that families play in decision-making within Bangladeshi society. In this cultural context, families primarily make decisions for patients, and physicians are expected to assist families in making appropriate medical decisions to safeguard patients’ ‘best interests’. Individual family members may have personal wishes and preferences, but collective familial interests always take precedence.

Farhat Moazam [39] argued that there is a cultural difference between Western and Eastern societies. A difference in healthcare practices compared to Western medical norms emphasises a patient’s right to be fully informed about his or her health status. In many cultures, particularly the U.K. and the U.S., patients have legal rights to be informed about the details of their health conditions, including the potential risks and benefits [40].

Such societies prioritise individual freedom, considering it a fundamental legal right [41]. In these secular cultures, when a competent patient is diagnosed with a serious medical prognosis, such as cancer, the doctors convey the truth to the patient without deception [21, 42]. Patients are encouraged to make decisions about their healthcare based on information provided by healthcare providers. In countries such as the U.K [43], the U.S [44], and many Western European countries [45], physicians are mandated to disclose all relevant information directly to their patients. This type of individualistic approach might be in contrast with the medical practices in Bangladesh, where family involvement in medical decision-making is a cultural reality.

Our research findings also indicate that families often request physicians to withhold true medical information from patients diagnosed with severe illnesses. This phenomenon can be attributed to the influence of family-centredness, filial piety and patriarchy in Bangladeshi Muslim culture. In this society, the family unit holds a leading role, with decisions made collectively and the interests of the family taking precedence over individual preferences.

Multi-generational living arrangements are common, with parents, adult children, and grandparents often residing under the same roof. Parents are highly respected, and junior family members receive care and support from elders. In traditional Bangladeshi patriarchal society, gender roles are typically defined by men leading families. The father is usually responsible for earning for the family, while women manage the household and nurture the children. Extended family dynamics involve all members in the decision-making process. Family autonomy, rather than patient autonomy, is the guiding principle, with decisions made collectively within the family structure.

Islamic collective cultural traditions further reinforce the importance of making decisions as a family unit [46]. The emphasis on family determination over individual autonomy in Bangladeshi Muslim society [47] can have implications for healthcare outcomes. This ethos is like many East Asian Confucian cultures [48]. The absence of patient autonomy and self-determination may impact the quality of care received and the overall healthcare experience for patients in this culture. Despite the lack of a legal framework and regulations supporting informed consent in Bangladesh, the BMDC’s guidelines are consistent with our findings. These guidelines instruct physicians to truthfully communicate the details of a patient’s medical condition to either the patient or his or her family members.

Our finding is consistent with the Confucian culture prevalent mostly in Mainland China [49], revealing that patients often perceive family members as advocates for their “best interests.” Ruiping Fan argued that physicians in East Asian societies, particularly Mainland China, are morally responsible for non-disclosure of the true medical prognosis if such disclosure may cause a severe burden on patients [49].

Another finding of this study is that families allowed physicians to disclose partial information to patients with their approval. The study revealed that the extent of disclosure is influenced by whether families authorise physicians to share information with patients. Additionally, the participants noted that physicians tend to provide information to patients incrementally as patients show signs of improvement during their recovery from illness.

This study identifies lying (deception) as a “therapeutic option” whereby the clinician intends to eliminate harm. Physicians are expected to disclose true information to families. Participants said that physicians discuss with the family how, when and to what extent the information is disclosed to the patient. In such cases, clinicians often use vague terms if necessary, such as telling a patient he or she has a tumour.

A survey of ninety physicians in twenty countries about attitudes towards revealing a ‘cancer’ diagnosis to patients reported that approximately 40% of oncologists from Africa, France, Hungary, Italy, Japan, Panama, Portugal and Spain chose to avoid using the word “cancer” while talking to patients. Physicians reported substituting words such as “swelling (e.g., tumour, growth, lump)” and ‘inflammation’, highlighting significant differences among these countries in terms of communicating information about life-threatening illnesses [50]. The key difference was that not all physicians in these countries disclosed the truth of the diagnosis to their families. Although recent empirical research in Muslim societies, particularly Lebanon, reported a trend towards greater acceptance of revealing a cancer diagnosis to the patient [51], a reluctance to practice full disclosure of information in such cases is still not unusual in many cultures.

Another major finding of this study is that the participants believed that directly disclosing the true medical diagnosis or information of a critically ill patient may be perceived as imposing a significant burden on the patient. The participants expressed the view that patients may struggle to cope with distressing news, leading to the practice of informing families when a severe disease is diagnosed. Applying contemporary ethical norms and medical practices prevalent in many countries, such as the U.K. and the U.S., may not align with Bangladesh’s collective culture.

Chattopadhyay and Simon [22] argued that culture plays a crucial role because it shapes the context in which individuals experience life and understand the moral significance of illness, suffering and death. They emphasised that culture profoundly influences how patients, families and physicians communicate and make decisions in end-of-life care. In several countries, much of Asia and the Middle East, withholding medical information is considered to be more humane and ethical [52]. A key reason participants mentioned was the protection of a patient’s emotional well-being and the reduction of psychological distress. In these cultures, the belief is that directly revealing distressing medical information to a patient may cause unnecessary anxiety, fear or hopelessness. Instead, by disclosing such information to the family, the burden of processing and managing the information can be shared collectively, potentially offering emotional support and comfort to the patient. In Ethiopian culture, for example, there are fears that patients can die from the shock of shocking news; therefore, the family handles determining how the information can be disclosed [53]. The participants also mentioned that patients are deceived and that deception should be an accepted medical practice in Bangladesh. Physicians deceive patients to follow the wishes of their families.

The participants also stated that a physician goes along with a family’s wishes if they are in a patient’s best interests. The participants thought that physicians accept that the family knows the patient well and are better appointed to anticipate their psychological state and how much information they can tolerate. The family wants to prevent a patient’s overburden, particularly if he or she is elderly, and wants to facilitate him or her with ‘dying in peace.’

This is a form of care, particularly for elderly end-stage family members. The concept of ‘death with dignity’, often cited in Western bioethics literature [54], is seldom practiced in Muslim-majority societies, such as Bangladesh. Here, ‘dying in peace’ is a common biomedical practice, especially for elderly patients [55]. This is because families and physicians do not want to overburden patients, as this may cause more psychological harm and the potential threat of dying. A reason for this disclosure relates to the nature of the disease—cancer—which is viewed by many as having a high chance of death. Revealing a diagnosis to a patient has been considered crucial, and the patient’s family believes that such disclosure would lead to a loss of hope, enhancing the patient’s anticipation of death.

Islamic beliefs and practices consistently remind Muslim believers to be prepared for death, as it cannot be delayed when time has passed [56]. One perspective highlighted here is that some might argue that informing patients of their terminal diagnosis might allow them to prepare for death in advance. This approach might enable patients to participate in medical decisions, reflect on their values and experience a more “dignified” death.

In Bangladesh, there may be patients who hold this viewpoint. In Islamic teachings, death is regarded as a natural and predetermined transition from earthly life to the hereafter. Death surrenders to the will of Almighty Allah. For Muslims, the ultimate hope for eternal life rests with the merciful God. Accepting death with patience, gratitude and faith in the divine plan while ‘death with dignity’ may place patients in control over the dying process. By approaching death with acceptance and trust in Allah’s wisdom, Muslims aim to achieve a peaceful transition to the afterlife. Preparing the soul for the next life, Muslim patients navigate the process of dying with grace and submission. For example, in Muslim societies, families may gather around terminally ill patients, recite prayers and provide comfort and support to ensure a peaceful transition.

This approach might reflect a profound respect for the Islamic principle of transitioning from earthly life to the hereafter with peace and spiritual fulfilment. When a family realises that a patient—particularly the elderly—is reaching his or her final hours, families may not allow clinicians to disclose shocking news to their patients so that death will occur with as little suffering as possible (dying in peace) [57]. This is a kind of morality (non-maleficence) that might prohibit families and physicians from fully disclosing a terminal diagnosis to the patient. It is common in other South Asian Muslim societies for extended families and physicians to avoid disclosing the diagnosis to a severely ill patient [39].

A significant finding highlights the prevalent practice of parents or legal guardians making decisions for minors under the age of eighteen in all medical treatments and interventions. In contemporary medical practice, there is an emphasis on involving competent adult patients in decisions regarding their medical care. In many cultures and legal systems, including those influenced by Islamic principles, minors under the age of eighteen lack the legal capacity to make independent decisions, particularly in matters related to healthcare.

Our observations revealed that parents or guardians accompanying minors received medical information and played the role of primary decision-makers. The participants in this study expressed a belief that parents are best positioned to determine what is in the best interests of their minor children, especially in the context of medical decision-making and healthcare treatment.

Bangladesh ratified rights-based United Nations (UN) conventions, including the UN Convention on the Rights of the Child, on June 26, 1990. Article 12 of this Convention underscores the significance of respecting children’s views and emphasises that adults should listen to and seriously consider them. The Convention also affirms that children possess fundamental human rights, including the rights to freedom of expression, to receive information of all kinds (Article 13), of thought, conscience and religion (Article 14) and to privacy (Article 16, paragraph 1). This highlights the importance of recognising children as individuals with rights and perspectives that should be taken into account in decision-making processes, including those related to their healthcare and well-being [58].

Belgian laws permit minors to make their own medical decisions until their physicians consider them sufficiently mature [59]. However, the legal framework governing medical practices may vary from country to country [60]. Like other South Asian societies, parents are typically consulted at all stages of decision-making for their children, as it is believed that children may make improper decisions regarding their health [61]. In countries, including those in Europe such as Bulgaria, Cyprus, Finland, France, Greece, Hungary, Italy, Malta, Romania and Slovakia, minors have no right to make independent decisions about their medical treatments until they reach the age of majority [62, 63].

Our findings also revealed that parents typically make medical decisions for their children (minors) and physicians often hesitate to directly inform minor patients, operating under the assumption that minors might not have the capacity to make appropriate medical decisions for themselves. Mark Cherry argues that minors may lack the cognitive or emotional maturity required to fully grasp complex medical information and potential outcomes [64]. In certain situations, Cherry contended that minors may be more inclined to make different, potentially “riskier decisions” due to emotional factors or an immature perception of the circumstances [64].

### Strengths and limitations of this study and suggestions for future research

A key strength of this study lies in its exploration of true medical disclosure, achieved by collecting first-hand experiences from patients, families and healthcare professionals within a Muslim society in Bangladesh. Another key strength is that these findings may help healthcare providers treat Bangladeshi diaspora patients in other countries. Additionally, this study’s findings have the potential to inform future research in regions such as traditional family structures and collectivistic societies [65].

Our study has several limitations. The sample size, although appropriate for the phenomenological approach utilised, was small and confined to a publicly funded hospital in the capital city of Bangladesh. This limited our ability to thoroughly explore our findings across various healthcare settings, such as public hospitals, private institutions, NGOs and international donor agencies throughout the country. Future research could address this limitation by including a more diverse range of participants from different healthcare settings.

Another significant limitation was the absence of involvement from health policymakers and public health officials in the study, which hindered a comprehensive analysis of law formulation and implementation. Including these stakeholders in future research could provide insights into legal regulations, cultural aspects of care and the dynamics of medical information disclosure. This approach could illuminate how patients, families and clinicians engage in decision-making processes related to medical disclosure and healthcare treatment.

## Conclusions and recommendations

The main finding of this phenomenological qualitative study is that it sheds light on the complex interplay of cultural norms, ethical considerations and legal obligations in the context of concealing true medical prognoses from patients. This study revealed that withholding true medical information from patients is perceived as an act in patients’ best interests because severe medical prognoses may be difficult for patients to tolerate and can cause significant health burdens.

This study also found that families determined whether and to what extent true information is disclosed, and clinicians acknowledged a family’s wishes if they ensured patients’ best interests. Although contemporary ethical norms and medical practices advocate for truth disclosure, the reality in Bangladeshi society often leans towards non-disclosure. This indigenous approach to medical practice mirrors the underlying socioeconomic reality, religious ethical values and collective cultural norms of Bangladesh, which seek to shield patients from potential harm by refraining from full disclosure.

This study offers two major suggestions, with policy and practical implications. First, the adoption of Western ethical norms has been limited in Bangladesh, with only a few physicians having formal training in bioethics. Formal training in medical ethics may help clinicians establish healthy relationships with patients and their families. The lack of formal bioethics training among healthcare providers may be a significant factor contributing to ambivalence towards non-disclosure culture within the healthcare settings in Bangladesh. It is suggested that the Bangladeshi government should arrange comprehensive medical ethics training for clinicians to promote healthy relationships with patients and their families.

Second, this study suggests that there is a need to enhance the role of patients within familial decision-making processes. Encouraging patients to engage in discussions with their family members and healthcare professionals can empower them to actively contribute to making familial medical decisions within the family unit. It is also essential to collaborate with clinicians to strike a balance between honouring patient preferences and maintaining familial involvement. Introducing a rights-based, patient-centred approach to medical decision-making in Bangladesh may not align with the collective consciousness of a family. Instead, a system of supportive, interdependent relationships among family members, including patients, is encouraged. Like other South Asian Muslim societies, this approach seeks to maintain a dynamic balance that preserves the important cultural values of duty and care within families while introducing the possibility for individual members to participate in their medical decision-making [39].

Cultural values that regard a family as the fundamental unit of society should be recognised and respected. These values emphasise a family’s primary role and the well-defined roles of each individual within it. It is recommended that the government of Bangladesh formulate a policy framework and regulations concerning true medical disclosure. Such a measure could guide clinicians in maintaining open discussions and communication with patients and their families about the extent and manner of medical information disclosure, potentially leading to improved healthcare outcomes.

## Electronic supplementary material

Below is the link to the electronic supplementary material.

Supplementary Material 1

## Data Availability

To protect the participants’ confidentiality, the data supporting this study’s findings are not publicly available. However, they may be available upon reasonable request from the corresponding author.

## Abbreviations

BMDC: Bangladesh Medical and Dental Council
GDP: Gross Domestic Product
NGO: Non-Governmental Organization
ShSMC: Shaheed Suhrawardy Medical College
U.K.: United Kingdom
UN: United Nations
U.S.: United States
