# Supplementary material for: Disclosure of true medical information: the case of Bangladesh
Source: BMC Med Ethics. 2024 Oct 17;25:112. doi: 10.1186/s12910-024-01115-y (PMC11483989; doi:10.1186/s12910-024-01115-y)
Supplement: Supplementary file 1 — Supplementary Material 1 [file 12910_2024_1115_MOESM1_ESM.docx]

**Role of Physicians, Patients, and Families in Making Medical Decisions in Bangladesh**

**Demographic Information**

- Name:
- Occupation/Profession:
- Age:
- Address:

**Interview Questions**

1. How is a patient treated if he or she is diagnosed with a disease?
2. When a patient is diagnosed with a minor illness, to whom is medical information provided/disclosed and why?
3. If a patient is diagnosed with a minor illness, how much medical information is given/provided to whom (patients or families) and why?
4. From whom and why is consent/decision sought to choose appropriate medical interventions/treatments for minor ill patients?
5. When a patient is diagnosed with a terminal illness (for example, cancer or other severe diseases), to whom is medical information disclosed/provided (patients or families) and why?
6. If a patient is diagnosed with a terminal illness (for example, cancer or other severe diseases), how much medical information is entrusted/provided to whom (patients or families) and why?
7. Who is asked for informed consent to choose appropriate medical interventions/treatments for terminally ill patients?
8. Who, and why, signs the informed consent form before major surgery on terminally ill patients?
9. What happens when family members disagree about which medical interventions/treatments to undertake? Who does what, and how are these disagreements resolved?
10. What happens when patients and family members disagree on which medical interventions/treatments to undertake? Who does what, and how are these disagreements resolved?
11. What happens if the family, the patient, and the physician disagree on the patient's treatment options? Who does what, and how are these disagreements resolved?
12. Do physicians have any role in deciding whether medical intervention is required to treat terminally ill patients?
13. How good is the doctor-patient relationship? Is there any patient mistrust of doctors? How can such mistrust be overcome?
14. Do you understand there is a difference between pre-pandemic and post-pandemic medical treatments used to treat patients with terminal illnesses?
